# Levels of Antioxidant Activity and Fluoride Content in Coffee Infusions of Arabica, Robusta and Green Coffee Beans in According to their Brewing Methods

**DOI:** 10.1007/s12011-017-0963-9

**Published:** 2017-02-22

**Authors:** J. Wolska, Katarzyna Janda, K. Jakubczyk, M. Szymkowiak, D. Chlubek, I. Gutowska

**Affiliations:** 1 Department of Biochemistry and Human Nutrition, Pomeranian Medical University in Szczecin, Broniewskiego 24 Street, 71-460 Szczecin, Poland; 2 Faculty of Applied Social Sciences and Resocialisation at Warsaw University, 00-503 Warsaw, Poland; 3 Department of Biochemistry, Pomeranian Medical University in Szczecin, Powstańców Wlkp. 71 Street, 70-111 Szczecin, Poland

**Keywords:** Coffee infusion, Antioxidant activity, DPPH, Fluoride content

## Abstract

Coffee is a rich source of dietary antioxidants, and this property links with the fact that coffee is one of the world’s most popular beverages. Moreover, it is a source of macro- and microelements, including fluoride. The aim of this work was to determine antioxidant activity of coffee beverages and fluoride content depending on different coffee species and conditions of brewing. Three species of coffee, arabica, robusta and green coffee beans obtained from retail stores in Szczecin (Poland) were analyzed. Five different techniques of preparing drink were used: simple infusion, french press, espresso maker, overflow espresso and Turkish coffee. Antioxidant potential of coffee beverages was investigated spectrophotometrically by DPPH method. Fluoride concentrations were measured by potentiometric method with a fluoride ion-selective electrode. Statistical analysis was performed using Stat Soft Statistica 12.5. Antioxidant activity of infusions was high (71.97–83.21% inhibition of DPPH) depending on coffee species and beverage preparing method. It has been shown that the method of brewing arabica coffee and green coffee significantly affects the antioxidant potential of infusions. The fluoride concentration in the coffee infusions changed depending, both, on the species and conditions of brewing, too (0.013–0.502 mg/L). Methods of brewing didn’t make a difference to the antioxidant potential of robusta coffee, which had also the lowest level of fluoride among studied species. Except overflow espresso, the fluoride content was the highest in beverages from green coffee. The highest fluoride content was found in Turkish coffee from green coffee beans.

## Introduction

Modern society, where people live faster and more intensely, makes stimulant beverages more and more popular. They remove fatigue and enhance level of concentration, which leads to better work efficiency and generally improve fettle [1]. Coffee, one of the most popular drinks in the world, is among those drinks and can have a stimulating effect on humans. For many people, especially in Western countries, the coffee drinking is a part of their lifestyle and an everyday habit. Morning cup of coffee is a daily routine for millions of people worldwide, about 40% of the world’s population starts a day that way [2, 3]. Coffee is consumed for various reasons; first of all, it is a stimulating drink because of its caffeine content; it benefits health and has excellent taste and aroma [3–5]. Its flavour, aroma and caffeine (1,3,7-trimethylpurine-2,6-dione) content play a role in its popularity, but coffee bean beverages are complex chemical mixture and contain more than a thousand different chemical compounds, such as carbohydrates, lipids, nitrogenous compounds, vitamins, minerals, alkaloids and phenolic compounds [6]. Different studies suggest that coffee consumption can reduce the risk of being affected by Alzheimer’s disease, Parkinson’s disease, heart disease, diabetes mellitus type 2, cirrhosis of the liver and some type of cancer [7–11]. Antioxidants have protective effect and neutralize free radicals, which are toxic byproducts of natural cell metabolism. Although, the human body produces endogenous antioxidants, it still depends on an adequate supply of exogenous antioxidants in the diet [11, 12]. Antioxidants are the most important health benefiting substances in coffee, and coffee drink is a rich source of them [11, 13]. Coffee beverages are also a source of minerals in the diet [6, 14, 15], including fluoride, but there are only a few works on it [16–18].

There is no literature data referencing the content and the effect of the method of preparing coffee infusion on the amount of fluoride and antioxidants content. An attempt was made to determine the correlation between the antioxidant activity and the content of fluoride in infusions.

## Material and Methods

### Material

Three species of coffee: arabica, robusta and green beans derived from retail shops in Szczecin (Poland) were analysed. Arabica and green coffee beans were from Antigua region in Guatemala, whereas robusta coffee came from India. For every type of brewing method 1.50 g of grinded coffee beans and 150.0 mL of water were used to prepare each infusion. Coffee beverages were taken for analyses after cooling them down to the room temperature. It was implied to make coffee drinks with similar variables as the one that is served at home or coffeehouse to make results easier to interpret in terms of daily life.

### Coffee Beverages Preparation

#### Simple Infusion

A known mass of coffee was placed inside the beaker, 150.0 mL of boiling hot water poured in and after 5 min coffee infusion samples were taken for analyses.

#### French Press

A French press, also called a press pot or a coffee plunger, device was used. The pot was placed on a flat surface; the plunger was pulled out, measured amount of coffee added and boiling hot water gently poured inside. Then the plunger was reinserted into the pot on the surface of the coffee beverage and plunged down after 5 min. Once the press plunger was put down a sample of coffee was taken for analyses.

#### Espresso Maker

A stove top espresso coffee pot device was used. Firstly, the lower chamber of the espresso pot was filled with 150.0 mL of cold water; secondly, into the middle section (the filter funnel) a known amount of ground coffee was placed. Funnel was put into a place in the pot and screwed on the upper section securely. Espresso maker was placed on the electric stove to allow boiling water to go through the filter with coffee to the upper section. Once all of the water transferred, the sample of coffee infusion was taken for analyses.

#### Overflow Espresso

An overflow coffee maker device was used. Grounded coffee beans were placed in a paper filter, water added to a tank and the machine turned on. When the coffee was ready, samples were taken for measurements.

#### Turkish Coffee

An original copper Turkish pot was used. Coffee beans were placed inside the pot, water added and brought to boiling on an electrical stove.

### Antioxidant Activity of Coffee Infusions

The antioxidant activity of samples was measured with spectrophotometric method using synthetic radical DPPH (2,2-diphenyl-1-picrylhydrazyl, Sigma) and spectrophotometer Agilent 8453UV. Ninety-six percent ethanol, 1 mL of 0.3 mM solution of DPPH in 96% ethanol, and 0.1 mL of the test sample were introduced into the vial in *v*/*v* ratio 29:10:1. The prepared solution after mixing was placed for 30 min in a dark place. During this time, the so-called A_0_ solution was prepared by mixing 96% ethanol and 0.3 mM solution of DPPH in *v*/*v* ratio 3:1. As a reference solution, 96% ethanol was used. Before the measurement, the vial contents were thoroughly mixed, poured into quartz cuvettes, and the spectral absorbance was immediately measured at 518 nm. All assays were performed in triplicate.

Antioxidant potential (antioxidant activity, inhibition) of tested solutions has been expressed by the percent of DPPH inhibition, using the formula:

$$ \%\kern0.5em \mathrm{inhibition}=\frac{A_{\mathrm{o}}-{A}_{\mathrm{s}}}{A_{\mathrm{o}}}\times 100 $$

where:

*A*_0_: absorbance of DPPH solution at 518 nm without tested sample
*A*_s_: absorbance of DPPH solution at 518 nm with tested sample

### Determination of Fluoride Content in Coffee Infusions

The coffee infusions were poured into a plastic tube, labelled and frozen at −20 °C until the determination of F^−^ levels. Fluoride concentrations in individual samples were measured by potentiometric method with a fluoride ion-selective electrode (Orion 9409 BN, Thermo Scientific, USA). One millilitre of sample was transferred to a plastic tube, and then 1.0 mL of TISAB II was added to this solution. After mixing, the potential difference of each sample was measured for 10 min; 5 min before the addition of the appropriate standard and 5 min after the addition. According to the work of Łukomska et al. [19], the fluoride content in samples was calculated based on the difference of potentials measured in each sample and the concentration of the added standard. The electrode has been calibrated using standard solutions. The correctness of the analytical procedure was controlled by determining the concentration of F^−^ in NaF solutions with known concentrations: 0.1, 1.0 and 10.0 mg/L (Orion Company, USA).

### Statistical Analysis

In all the experiments, three samples were analysed, and all the assays were carried out at least in triplicate. Statistical analysis was performed using Stat Soft Statistica 12.5 and Microsoft Excel 2007. The results are expressed as mean values and standard deviation (SD). The distribution of results for individual variables was obtained with the Shapiro–Wilk *W* test. For fluoride content results, as most of the distributions deviated from the normal Gaussian distribution, non-parametric tests were used for further analyses (Wilcoxon test, *U* Mann–Whitney test). For percent of inhibition, DPPH were used one-way analysis of variance (ANOVA), Tukey post-hoc test. Correlation analysis was performed by Spearman rho coefficient. Differences were considered significant at *p* ≤ 0.05.

## Results

Antioxidant activity of infusions was high (71.97–83.21% inhibition of DPPH) and dependent on the species of coffee used and the condition of brewing.

**Fig. 1:**
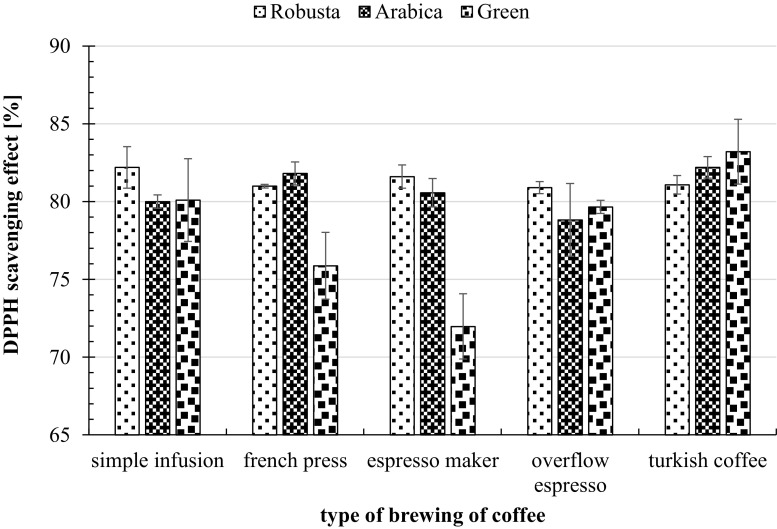
Antioxidant activity of infusions in dependence on conditions for brewing arabica, robusta and green coffee. * Indicates significant differences at *p* ≤ 0.05

However, significant differences were found only between green coffee and arabica, and green coffee and robusta, when french press and espresso maker were used as brewing methods. No significant differences in antioxidant potential between different species of coffee were measured while using simple infusion, coffee maker and Turkish method of brewing (Fig. 1).

It has been shown, that the method of brewing for arabica and green coffee significantly affects the antioxidant potential of infusions. That did not apply to different ways of brewing for robusta coffee (Table 1).

**Table 1:** Differences in coffee antioxidant activity (% inhibition of DPPH) depending on the species and the method of preparing the infusion

Methods of preparing the infusion	Coffee species		
	Arabica	Robusta	Green coffee
Simple infusion vs French press	ns	ns	ns
Simple infusion vs espresso maker	ns	ns	*
Simple infusion vs overflow espresso	ns	ns	ns
Simple infusion vs Turkish coffee	ns	ns	ns
French press vs espresso maker	ns	ns	ns
French press vs overflow espresso	*	ns	ns
French press vs Turkish coffee	ns	ns	*
Espresso maker vs overflow espresso	ns	ns	*
Espresso maker vs Turkish coffee	ns	ns	*
Overflow espresso vs Turkish coffee	*	ns	ns

*ns* not significant

*Indicates significant differences at *p* ≤ 0.05

**Fig. 2:**
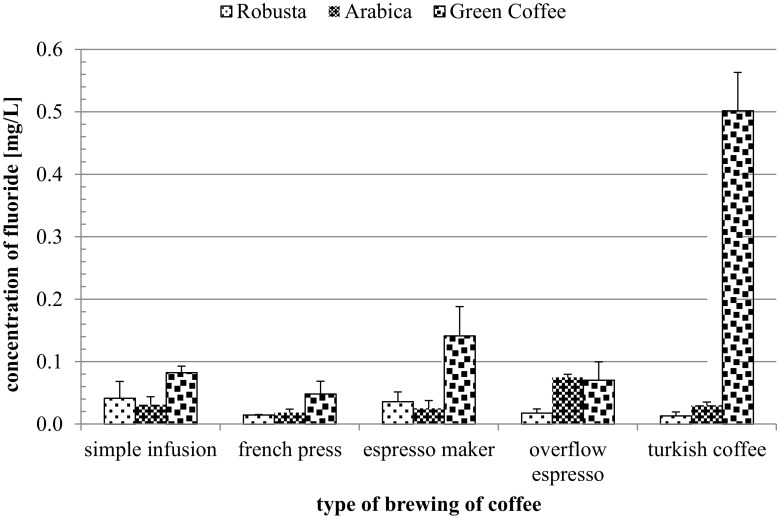
Fluoride content in infusions in dependence on conditions for brewing arabica, robusta and green coffee. * Indicates significant differences at *p* ≤ 0.05

The fluoride concentration in the coffee infusions changed depending both on the species and conditions of brewing, too. Except overflow espresso, the fluoride content was the highest in beverages from green coffee. The highest fluoride content was found in Turkish coffee from green coffee beans (Fig. 2). Significant differences were found between arabica coffee and robusta when Turkish method and overflow espresso were used as brewing methods. Except overflow espresso, for arabica vs green coffee significant differences were found or all brewing methods. Significant differences were found between robusta vs green coffee when all methods of brewing were used (Fig.2).

The differences in fluoride content in coffee depending on the species and the method of preparing the infusions are presented in Table 2. Statistically significant differences in fluoride content have been noticed for arabica coffee beans in the following infusions: french press vs espresso maker and french press vs Turkish coffee. Statistically significant differences in fluoride content have been noticed for robusta coffee beans in the following infusions: simple infusion vs french press, simple infusion vs overflow espresso, simple infusion vs Turkish coffee, french press vs espresso maker and espresso maker vs Turkish coffee. Statistically significant differences in fluoride content have been noticed for green coffee beans in the following infusions: simple infusion vs french press, simple infusion vs espresso maker, simple infusion vs overflow espresso, french press vs espresso maker and espresso maker vs overflow espresso (Table 2). Conducted experiments did not unequivocally answer the question, if the type of brewing method affects significantly the fluoride content in coffee beverages. As it turned out the content of fluorides in coffee infusions could have also depended on types of used coffee beans.

**Table 2:** Differences in fluoride content in coffee depending on the species and the method of preparing the infusion

Methods of preparing the infusion	Coffee species		
	Arabica	Robusta	Green coffee
Simple infusion vs French press	ns	*	*
Simple infusion vs espresso maker	ns	ns	*
Simple infusion vs overflow espresso	ns	*	*
Simple infusion vs Turkish coffee	ns	*	ns
French press vs espresso maker	*	*	*
French press vs overflow espresso	ns	ns	ns
French press vs Turkish coffee	*	ns	ns
Espresso maker vs overflow espresso	ns	ns	*
Espresso maker vs Turkish coffee	ns	*	ns
Overflow espresso vs Turkish coffee	ns	ns	ns

*ns* not significant

*Indicates significant differences at *p* ≤ 0.05

Correlation analysis was used to explore the relationships amongst the antioxidant activities and fluoride content in coffee infusion (Table 3). The antioxidant activity exhibited a diverse correlation (*p* < 0.05) with fluoride content in coffee infusions. Negative correlations were in infusion prepared by french press and overflow espresso (−0.64*) and espresso maker (−0.57*). The strongest and positive correlation (0.83*) was in Turkish coffee.

**Table 3:** Spearman rho correlation coefficients (R) between antioxidant acivity and fluoride content in coffee infusions

Preparing method of coffee infusion	Correlation coefficient (R)
Simple infusion	−0.07
French coffee	−0.64*
Espresso maker	−0.57*
Overflow espresso	−0.64*
Turkish coffee	0.83*

*Correlation is significant at *p* ≤ 0.05

## Discussion

Arabica coffee (*Coffea arabica*) and robusta coffee (*Coffea canephora*) have different growing conditions and chemical composition, and thereafter organoleptic properties (particularly taste and smell) of their beans and infusions are also different [20]. Fărcaş et al. [21] show that the infusions of robusta coffee (solid-liquid extraction, 1 g of ground coffee, 100 mL of hot deionized water, 2 min) have antioxidant potential of 43.63% inhibition of DPPH and infusion of arabica coffee only 36.18%, while our study showed that the antioxidant potential may be much higher. Depending on the brewing method, level of antioxidant potential ranged from 78.92 to 82.2% inhibition of DPPH for arabica and from 80.90 to 82.2% inhibition of DPPH (no significance of differences) for robusta. Our research has indicated, that, depends on brewing method, the greatest difference in antioxidant potential was found in green coffee infusions (71.97–83.21%). Ramadan-Hassanien [22] has studied and measured antioxidant potential of instant coffee, Turkish coffee and cappuccino, using the same brewing method (2 g coffee, 200 mL of boiling water, 5 min.). Cappuccino had the highest antioxidant activity (66.0% inhibition of DPPH), second was Turkish coffee (33.2%) and third was instant coffee (14.0% inhibition of DPPH). The study clearly confirms that the type of coffee has a significant impact on the antioxidant potential level of infusions. Thermal treatment of coffee beans (roasting process) also affects the level of antioxidant potential. Roasting process transforms the chemical and biological properties of coffee beans and increases its antioxidant activity [23–26]. Finally, antioxidant activity of roasted coffee beans depends on their different composition. In addition to the phenolic compounds occurring in coffee, which are partially destroyed by the roasting process, other antioxidant compounds, such as melanoidins, may be formed, and so it is possible to maintain or increase antioxidant activity. However, as the intensity of roasting increases, the greater destruction of the phenolic compounds may not be compensated for by the formation of other compounds. Hence, coffees originating from light roasting show greater antioxidant capacity due to the greater polyphenol content [20]. According to Hecimovic et al. [25] roasted coffee beans exhibited higher antioxidant capacity than green coffee beans, and intensified coffee roasting resulted in a decrease of its antioxidant potential. However, our results have shown that the highest antioxidant capacity was measured in unroasted coffee beans infusion. It should be emphasized, that in our study, five different ways of brewing coffee were used, and it is possible that it was the factor determining the highest antioxidant potential of green coffee beans. In order to verify our results with other studies, further extended analysis of that subject is needed. According to Vince et al. [27], coffee beverages could reduce the consequences of oxidative stress in the organism. Our results confirm that infusions of coffee are a rich source of antioxidant compounds.

Beverages, mainly coffee and tea, are the main source of fluoride in our diet [18, 28–30]. Studies have shown that, regardless of the method of brewing, the highest fluorine content was recorded in green coffee infusions. This might suggest that the process of coffee roasting may cause the formation of low soluble fluoride compounds, which reduces its ability to go into solution during brewing. There are no documented studies on the effects of the extraction time for the fluorine content in the infusions of coffee. However, there are some publications on the effects of tea brewing time on the content of this element in the infusion. Many authors of these studies have clearly proved that the longer the tea brewing time (for 120 min), the higher the fluorine content of the infusion (depending on the type of tea 2–3 times higher) [31–33]. Although the fluorine content in coffee is not high, the studies on animals have shown that plasma fluoride (F) concentrations are higher by up to 100% when F is administered in coffee or a caffeine solution compared with when it is administered in water [34]. The authors noted no differences among the groups in the renal or extrarenal (skeletal) clearances of F, which suggested that the higher plasma F concentrations in the coffee groups may have been due to a slight and transient increase in absorption rate. The results indicated that decaffeinated coffee and caffeine had no effect on F metabolism, whereas caffeinated coffee appeared to increase the initial absorption rate [34]. The results of these studies were also confirmed by Chan et al. [35] who noted that at the same time intragastric administration of sodium fluoride and coffee resulted in a significantly higher (P less than 0.01) plasma fluoride level than intake of the same amount of fluoride with water. The same result was obtained when coffee was substituted with an equivalent amount of caffeine [35]. These studies, in the context of our results, suggest that despite the low fluoride content in our coffee infusions, absorption of this element can be high. This finding could help explain the variations in the incidence of dental fluorosis among people living in optimally fluoridated communities [35]. Many in vivo studies confirmed protective effects of non-enzymatic antioxidant compounds such as vitamins and non-vitamin oxidants against the consequences of exposure to fluoride [36–40]. Coffee beans infusions are a valuable source of antioxidants, but their contents depend on the brewing process and the type of coffee beans used for brewing. The highest antioxidant potential was detected in green coffee infusion, but it also contained the high level of fluoride. Robusta coffee drink, because of its high antioxidant status, which was independent of the method of brewing, and the lowest level of fluoride, seems to be a healthier choice. Further studies on using antioxidants, both in vivo and in vitro, need to be undertaken for a better intoxication prevention in cases of increased exposure to the fluorine.

## References

[1] Żukiewicz-Sobczak W, Krasowska E, Sobczak P, Horoch A, Wojtyła A, Piątek J (2012). Effect of coffee consumption on the human organism. Medycyna Ogólna i Nauki o Zdrowiu.

[2] Mussatto SI, Machado EMS, Martins S, Teixeira JA (2011). Production, comnsumption, and aplication of coffee and its industrial residues. Food Bioprocess Technol.

[3] Oliveira M, Casal S, Morais S, Alves C, Dias F, Ramos S, Mendes E, Delerue-Matos C, Oliveira BPP (2012). Intra- and interspecific mineral composition variability of commercial coffees and coffee substitutes. Contribution to mineral intake. Food Chem.

[4] Grembecka M, Malinowska E, Szefer P (2007). Differentiation of market coffee and its infusions in view of their mineral composition. Sci Total Environ.

[5] Butt MS, Sultan MT (2011). Coffee and its consumption: benefits and risks. Crit Rev Food Sci Nutr.

[6] Farah A (2012) Coffee constituents. In: Chu Y-F (ed) In Coffee: Emerging Health Effects and Disease Prevention, 1st edn. John Wiley & Sons, Inc. Published 2012 by Blackwell Publishing Ltd. http://www.ift.org/~/media/Knowledge%20Center/Publications/Books/Samples/IFTPressBook_Coffee_PreviewChapter.pdf. Accessed 30 Oct 2016

[7] Ascherio A, Weisskopf MG, O’Reilly EJ, McCullough ML, Calle EE, Rodriguez C, Thun MJ (2004). Coffee consumption, gender, and Parkinson’s disease mortality in the cancer prevention study II cohort: the modifying effects of estrogen. Am J Epidemiol.

[8] Molloy JW, Calcagno CJ, Williams CD, Jones FJ, Torres DM, Harrison SA (2012). Association of coffee and caffeine consumption with fatty liver disease, nonalcoholic steatohepatitis, and degree of hepatic fibrosis. Hepatology.

[9] Higdon JV, Frei B (2006). Coffee and health: a review of recent human research. Crit Rev Food Sci Nutr.

[10] Bae JH, Park JH, Im SS, Song DK (2014). Coffee and health. Integr Med Res.

[11] Fiedor J, Burda K (2014). Potential role of carotenoids as antioxidants in human health and disease. Nutrients.

[12] Saikat S, Chakraborty R, Sridhar CY, Reddy SR, Biplab D (2010). Free radicals, antioxidants, diseases and phytomedicine: current status and future prospect. Int J Pharm Sci Rev Res.

[13] Sroka Z, Janiak M, Dryś A (2015). Antiradical activity and amount of phenolic compounds in extracts obtained from some plant raw materials containing methylxanthine alkaloids. Herba Pol.

[14] Oliveira M, Ramos S, Delerue-Matos C, Morais S (2015). Espresso beverages of pure origin coffee: Mineral characterization, contribution for mineral intake and geographical discrimination. Food Chem.

[15] Özdestan Ö (2014). Evaluation of bioactive amine and mineral levels in Turkish coffee. Food Res Int.

[16] Tokalioğlu S, Kartal U, Turk Ş (2004). Determination of fluoride in various samples and some infusions using a fluoride selective electrode. J Chem.

[17] GJ M, Ong B, Quinlan K, Riah A, Thomas J (1980). The determination of fluorine in coffee and tea using a microprocessor coupled with a fluoride ion-selective electrode. J Food Technol.

[18] Warren DP, Henson HA, Chan JT (1996). Comparison of fluoride content in caffeinated, decaffeinated and instant coffee. Fluoride.

[19] Łukomska A, Jakubczyk K, Maciejewska D, Baranowska-Bosiacka I, Janda K, Goschorska M, Chlubek D, Bosiacka B, Gutowska I (2015). The fluoride content of yerba mate depending on the country of origin and the conditions of the infusion. Biol Trace Elem Res.

[20] Vignoli JA, Viegas MC, Bassoli DG, de Toledo Benassi M (2014). Roasting process affects differently the bioactive compounds and the antioxidant activity of arabica and robusta coffees. Food Res Int.

[21] Fărcaş AC, Socaci SA, Bocăniciu J, Pop A, Tofană M, Muste S (2014). Evaluation of biofunctional compounds content from brewed cofee. Bulletin UASVM Food Science and Technology.

[22] Ramadan-Hassanien MF (2008). Total antioxidant potential of juices, beverages and hot drinks consumed in Egypt screened by DPPH in vitro assay. Grasas Aceites.

[23] Jeong JH, Jeong HR, Jo YN, Kim HJ, Lee U, Heo HJ (2013). Antioxidant and neuronal cell protective effects of columbia arabica coffee with different roasting conditions. Prev Nutr Food Sci.

[24] Sánchez-González I, Jiménez-Escrig A, Saura-Calixto F (2005). In vitro antioxidant activity of coffees brewed using different procedures (Italian, espresso and filter). Food Chem.

[25] Hecimović I, Belšcak-Cvitanović A, Horzić D, Komes D (2011). Comparative study of polyphenols and caffeine in different coffee varieties affected by the degree of roasting. Food Chem.

[26] del Castillo MD, Ames JM, Gordon MH (2002). Effect of roasting on the antioxidant activity of coffee brews. J Agric Food Chem.

[27] Vicente SJV, Queiroz YS, Gotlieb SLD, da Silva Torres EAF (2014). Stability of phenolic compounds and antioxidant capacity of regular and decaffeinated coffees. Braz Arch Biol Technol.

[28] Jędra M, Urbanek-Karłowska B, Gawarska H, Sawilska-Rautenstrauch D (2006). Fluoride content of soft drinks produced in Poland. Rocz Panstw Zakl Hig.

[29] Gupta P, Sandesh N (2012). Estimation of fluoride concentration in tea infusions, prepared from different forms of tea, commercially available in Mathura city. J Int Soc Prev Community Dent.

[30] Malinowska E, Inkielewicz I, Czarnowski W, Szefer P (2008). Assessment of fluoride concentration and daily intake by human from tea and herbal infusions. Food Chem Toxicol.

[31] Maleki A, Abulmohammadi P, Teymouri P, Zandi S, Daraei H, Mahvi AH, Shahsawari S (2016). Effect of brewing time and water hardness on fluoride release from different Iranian teas. Fluoride.

[32] Zhu JJ, Tang ATH, Matinlinna JP, Tsoi JKH, Hägg U (2013). Potentiometric determination of fluoride release from three types of tea leaves. Int J Electrochem Sci.

[33] Zerabruk S, Chandravanshi BS, Feleke Zewge F (2010). Fluoride in black and green tea (*Camellia sinensis*) infusions in Ethiopia: measurement and safety evaluation. Bull Chem Soc Ethiop.

[34] Chen X, Whitford GM (1994). Lack of significant effect of coffee and caffeine on fluoride metabolism in rats. J Dent Res.

[35] Chan JT, Qui CC, Whitford GM, Weatherred JG (1990). Influence of coffee on fluoride metabolism in rats. Proc Soc Exp Biol Med.

[36] Guney M, Oral B, Demirin H, Karahan N, Mungan T, Delibas N (2007). Protective effects of vitamins C and E against endometria damage and oxidative stress in fluoride intoxication. Clin Exp Pharmacol Physiol.

[37] Stawiarska-Pieta B, Paszczela A, Grucka-Mamczar E, Szaflarska-Stojko E, Birkner E (2009). The effect of antioxidative vitamins A and E and coenzyme Q on the morphological picture of the lungs and pancreata of rats intoxicated with sodium fluoride. Food Chem Toxicol.

[38] Stawiarska-Pięta B, Bielec B, Birkner K, Birkner E (2012). The influence of vitamin E and methionine on the activity of enzymes and the morphological picture of liver of rats intoxicated with sodium fluoride. Food Chem Toxicol.

[39] Stawiarska-Pięta B, Kubina R, Kabała-Dzik A (2015). The effect of propolis against toxicity of sodium fluoride in vitro. Post Fitoter.

[40] Inkielewicz-Stepniak I, Czarnowski W (2010). Oxidative stress parameters in rats exposed to fluoride and caffeine. Food Chem Toxicol.

